# The Role of Sex and Depression on Cognitive Impairment Risk in a Longitudinal Study of Older Adults

**DOI:** 10.1093/geroni/igaf122.102

**Published:** 2025-12-31

**Authors:** Lauren Rutter, Lucas Hamilton, Skylar Wilson, Anne Krendl, Brea Perry

**Affiliations:** Indiana University Bloomington, Bloomington, Indiana, United States; Augustana University, Sioux Falls, South Dakota, United States; Indiana University Bloomington, Bloomington, Indiana, United States; Indiana University Bloomington, Bloomington, Indiana, United States; Indiana University Bloomington, Bloomington, Indiana, United States

## Abstract

Depression and biological sex independently influence Alzheimer’s disease and related disorders (ADRD) risk, yet their interaction is underexplored. This study examines whether depression differentially impacts impairment risk for men and women in a large, longitudinal sample. Data were obtained from the National Alzheimer’s Disease Coordinating Center Uniform Data Set (N = 26,362, 59.3% women). Depression status was determined using the Geriatric Depression Scale (GDS≥5), and impairment was defined as progression to mild cognitive impairment or dementia. Impairment status was decided by either a consensus panel or a single physician. Cox proportional-hazards models tested the effects of depression, sex, and their interaction on impairment risk, controlling for age and APOEε4 status. Results showed significant main effects of depression, sex, age, and APOEε4 on impairment risk, and a significant interaction of depression and sex (p<.01). Depression increased impairment risk by 74% in men (HR = 1.74, p<.001) and 106% in women (HR = 2.06, p<.001). While men had a higher overall hazard ratios for impairment, depression disproportionately accelerated impairment risk in women. This difference may stem from biological mechanisms such as hormonal influences and neuroinflammation or psychosocial factors including caregiving roles and stress exposure. Our current work is exploring sex differences in perceived stress, and the potential mediating role of perceived stress in predicting impairment. Findings highlight the importance of considering sex differences when considering depression as a risk factor for cognitive decline. Targeted interventions addressing depression in women may be particularly effective in mitigating dementia risk and promoting cognitive resilience in aging populations.

